# Effectiveness of virtual reality–based intervention for adolescent depressive disorder: a randomized controlled trial protocol

**DOI:** 10.3389/fpsyg.2026.1845011

**Published:** 2026-07-07

**Authors:** Junqiang Zhao, Zhongnan Gong, Zhan Yin, Qianhui Zhai, Linlin Luo, Lina Wang, Fang Yan, Ying Niu, Junlei Zhang

**Affiliations:** 1 The First Affiliated Hospital of Henan Medical University, Xinxiang, Henan, China; 2 School of Nursing, Henan Medical University, Xinxiang, Henan, China; 3 School of Medical Engineering, Henan Medical University, Xinxiang, Henan, China; 4 The Second Affiliated Hospital of Henan Medical University, Xinxiang, Henan, China

**Keywords:** adolescent depressive disorder, cognitive function, heart rate variability, randomized controlled trial, virtual reality

## Abstract

**Background:**

The prevalence of depressive disorders among adolescents has been increasing in recent years, significantly impairing emotional development and social functioning. Although conventional treatments, including pharmacotherapy and psychotherapy, have demonstrated effectiveness, limitations such as poor adherence and restricted therapeutic settings remain. Virtual reality (VR) technology, which provides immersive and interactive environments, has shown promising potential in the treatment of mental disorders. However, high-quality evidence regarding its effectiveness in adolescents with depressive disorders remains limited.

**Methods:**

Protocol version 1.0, January 2026. This study is designed as a single-center, parallel-group randomized controlled trial. A total of 72 adolescents with depressive disorder who meet the eligibility criteria will be recruited and randomly assigned in a 1:1 ratio to either the intervention group or the control group. Participants in the control group will receive usual care, while those in the intervention group will receive additional VR-based intervention for 4 weeks (three sessions per week, approximately 25 min per session). Assessments will be conducted at baseline (T0), post-intervention (T1), and follow-up (T2). The primary outcome is the change in the 24-item Hamilton Depression Rating Scale (HAMD-24) score from T0 to T1. Secondary outcomes include the Hamilton Anxiety Rating Scale-14 (HAMA-14), Children’s Depression Inventory (CDI), Montreal Cognitive Assessment (MoCA), and heart rate variability (HRV) parameters (SDNN, RMSSD, LF, HF, and LF/HF). Data will be analyzed using linear mixed-effects models under the intention-to-treat principle.

**Discussion:**

This study will systematically evaluate the clinical effectiveness of VR-based intervention in adolescents with depressive disorder, as well as its impact on emotional symptoms, cognitive function, and autonomic nervous system activity. The findings are expected to provide evidence-based support for the application of VR technology in adolescent mental health care.

**Trial registration:**

https://www.chictr.org.cn, ChiCTR2500114846.

## 1 Background

Depressive disorder is a common mental health condition characterized by persistent low mood (World Health Organization, 2023), loss of interest, and reduced energy. The global burden of depression continues to rise, with more than 300 million individuals affected worldwide, leading to substantial impairment in quality of life and significant socioeconomic costs. Notably, the peak onset of depression occurs during late adolescence and early adulthood, making this population particularly vulnerable.

In China, rapid social changes have exposed adolescents to increasing academic pressure, social challenges, and developmental stressors, contributing to a rising prevalence of mental health problems (Wu et al., 2022). Adolescent depressive disorder presents with distinct clinical features, including emotional instability, irritability, and somatic complaints, and is often associated with a higher risk of impulsive behaviors and suicide. Importantly, adolescence represents a critical period of neurodevelopment, during which cognitive control, emotional regulation, and social cognition are not yet fully matured (Menculini et al., 2024). This developmental vulnerability may predispose adolescents to maladaptive cognitive patterns, such as negative automatic thoughts and rumination, which can exacerbate and maintain depressive symptoms (Nigg, 2017). In addition, emerging evidence (Zhao et al., 2023; Li X. et al., 2025), suggests that adolescents with depression frequently exhibit cognitive impairments, particularly in memory, executive function, and attention (Schumacher et al., 2024).

Current treatment strategies for adolescent depression primarily include pharmacotherapy and psychotherapy. While these approaches have demonstrated efficacy, pharmacological treatments may be associated with adverse effects, and psychotherapeutic interventions often face challenges related to limited accessibility, low adherence, and restricted therapeutic settings. Therefore, there is a need for innovative and engaging interventions that can enhance treatment effectiveness and patient participation.

Virtual reality (VR) technology, as an advanced human–computer interaction tool, enables users to experience immersive and interactive virtual environments (Riva and Serino, 2020). It has been widely applied in medical education (Baghaei et al., 2021), surgical simulation, and rehabilitation (Paul et al., 2022; Pedram and Piatkowski, 2025), and has shown particular promise in the field of mental health. Compared with traditional interventions, VR allows for the creation of controlled, customizable, and safe therapeutic scenarios (Clayborne et al., 2019), thereby enhancing patient engagement and treatment adherence. Previous studies have demonstrated the effectiveness of VR-based interventions in various psychiatric conditions, including post-traumatic stress disorder, specific phobias, and anxiety disorders (Menculini et al., 2024; Wang Z. et al., 2025). Moreover, meta-analytic evidence (Kampmann et al., 2016) suggests that VR interventions are effective in improving mental health outcomes in adults.

However, despite these encouraging findings, the application of VR in adolescent mental health remains limited (Anderson et al., 2013; Blanco et al., 2024). Existing evidence is largely derived from small-scale studies or extrapolated from adult populations, and high-quality randomized controlled trials specifically targeting adolescents with depressive disorder are still scarce (Rothbaum et al., 1999).

Virtual reality interventions may not only affect psychological symptoms but also influence physiological processes related to autonomic nervous system function. Heart rate variability (HRV), a widely used index of autonomic regulation, reflects the dynamic balance between sympathetic and parasympathetic nervous system activity (Agorastos et al., 2023). According to the neurovisceral integration model (Sgoifo et al., 2015), reduced HRV is associated with emotion regulation difficulties, a finding consistently observed in patients with major depressive disorder. Decreased parasympathetic activity—reflected by lower HRV indices such as RMSSD and high-frequency (HF) power—has been shown to correlate with greater depression severity and reduced emotional flexibility (Agorastos et al., 2023).

VR-based interventions may improve autonomic nervous system regulation through multiple pathways. Immersive and interactive virtual environments can promote relaxation, reduce psychological stress, and enhance emotion regulation, thereby facilitating parasympathetic activation and restoring autonomic balance (Velana et al., 2022). Previous studies have shown that (Robbemond et al., 2026) VR-based relaxation and mental health interventions reduce anxiety and stress responses, and these effects may be associated with positive changes in autonomic nervous system activity. Through these mechanisms, virtual reality interventions may increase vagally mediated heart rate variability indices and improve sympathovagal balance. Therefore, we hypothesize that VR interventions may improve autonomic nervous system regulation in adolescents.

## 2 Objectives

The primary objective of this study is to evaluate the clinical effectiveness of a virtual reality (VR)–based intervention in adolescents with depressive disorder.

The specific objectives are as follows:

(1) To assess the effect of the VR-based intervention on the severity of depressive symptoms in adolescents with depressive disorder;
(2) To examine the impact of the intervention on anxiety symptoms and cognitive function;
(3) To explore changes in selected physiological indicators before and after the intervention as supplementary measures of treatment effectiveness.

## 3 Methods

### 3.1 Study design

This study is a single-center, parallel-group randomized controlled trial designed to evaluate the clinical effectiveness of a virtual reality (VR)–based intervention in adolescents with depressive disorder. Eligible participants will be randomly assigned in a 1:1 ratio to either the intervention group or the control group.

**Figure 1:**
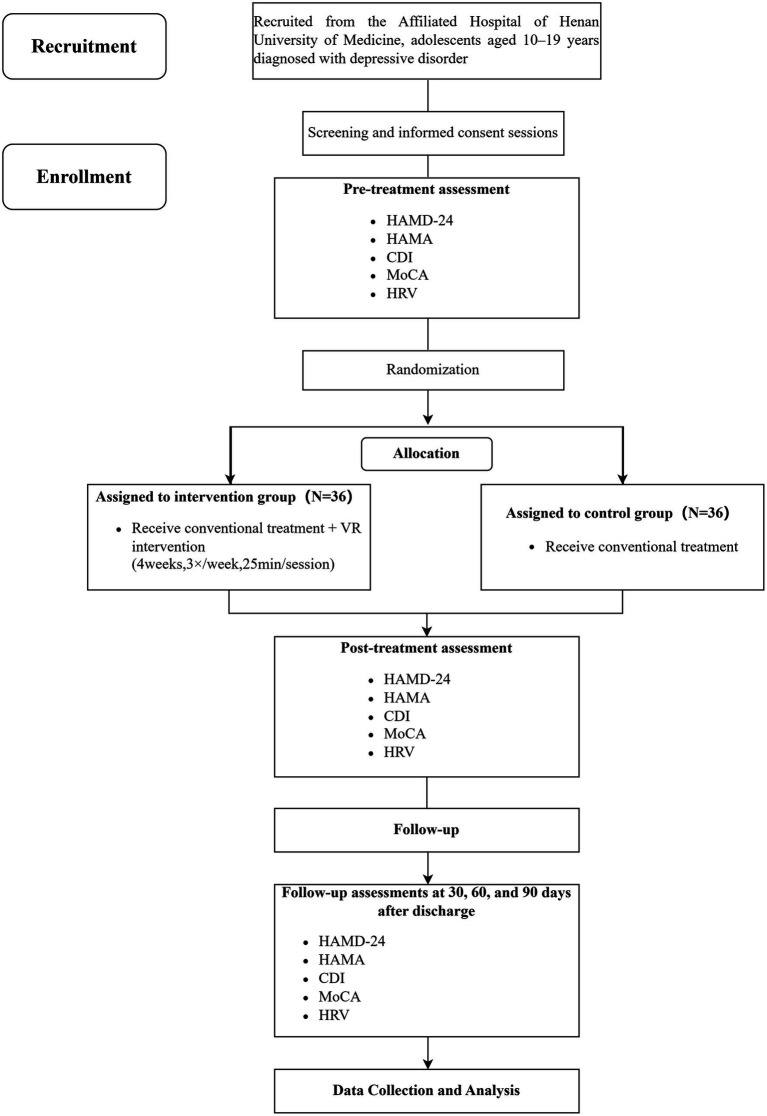
Flowchart.

Assessments will be conducted at baseline (T0), immediately after the intervention (T1), and at follow-up (T2). The study protocol has been developed in accordance with the SPIRIT (Standard Protocol Items: Recommendations for Interventional Trials) guidelines (Figure 1).

### 3.2 Study setting

Participants will be recruited from the Department of Child and Adolescent Psychiatry at Henan Provincial Mental Hospital. All assessments and interventions will be conducted in a standardized clinical setting within the hospital.

### 3.3 Participants

#### 3.3.1 Inclusion criteria

Participants must meet all of the following criteria:

1. Aged 10–19 years;
2. Diagnosed with depressive disorder according to the International Classification of Diseases, 10th Revision (ICD-10) by two qualified psychiatrists;
3. Receiving stable pharmacological treatment, with no changes in primary antidepressant dosage within the past two weeks;
4. Able to understand study procedures and comply with assessments and interventions;
5. Provided written informed consent from both the participant and their legal guardian.

#### 3.3.2 Exclusion criteria

Participants will be excluded if they meet any of the following criteria:

1. Presence of severe neurological disorders, organic brain diseases, or a history of epilepsy;
2. Comorbid severe psychiatric disorders (e.g., schizophrenia or bipolar disorder);
3. Severe physical illness or unstable vital signs;
4. Significant motion sickness or inability to tolerate VR equipment;
5. Participation in other interventional clinical studies during the study period.

### 3.4 Sample size

The sample size for this study was estimated based on data from Zeng et al. (2023). In that study, the post-intervention HAMD score was 21.30 ± 5.03 in the intervention group and 25.64 ± 5.39 in the control group, yielding an expected between-group difference (*δ*) of 4.34 points and an estimated pooled standard deviation (*σ*) of approximately 5.21. Using a two-sided significance level of *α* = 0.05 and a statistical power of 90% (*β* = 0.10), the required sample size was calculated using the formula for comparing the means of two independent groups. A minimum of 32 participants per group was estimated. Accounting for a potential dropout rate of 10%, the final sample size was increased to 36 participants per group, resulting in a total target sample size of 72 participants.

### 3.5 Randomization and blinding

Randomization will be performed using a computer-generated random sequence by an independent researcher who is not involved in participant recruitment or intervention delivery. Participants will be allocated to the intervention or control group in a 1:1 ratio.

Outcome assessors and data analysts will be blinded to group allocation to minimize assessment and analysis bias. Due to the nature of the intervention, participants and intervention providers cannot be blinded.

### 3.6 Interventions

#### 3.6.1 Control group

Participants in the control group will receive standard inpatient psychiatric care as provided by the Department of Child and Adolescent Psychiatry. Standard care includes pharmacological treatment prescribed by the attending psychiatrist, routine nursing care, symptom monitoring, safety assessments, medication management, and supportive communication as part of usual inpatient care. Pharmacological treatment will be adjusted by the attending psychiatrist based on clinical needs and will not be influenced by the research team.

Supportive communication is routinely provided by psychiatrists and psychiatric nurses during daily clinical interactions, with an emphasis on emotional support, treatment adherence, encouragement of coping skills, and risk assessment. However, participants will not receive any structured psychological therapy during the study period. To minimize potential confounding factors, both groups will receive the same standard inpatient psychiatric care throughout the study. The only difference between the two groups is that participants in the intervention group will additionally receive a virtual reality-based intervention. Standardized case report forms will be used to document medication use, medication adjustments, psychiatric consultations, and other clinically relevant interventions during the study period in order to monitor treatment exposure and potential co-interventions.

#### 3.6.2 Intervention group

Participants in the intervention group will receive a VR-based intervention in addition to usual care. The intervention will be delivered over a period of 4 weeks, with three sessions per week, and each session lasting approximately 25 min.

#### 3.6.3 VR intervention content and equipment

The VR program consisted of two modules: relaxation training and interactive scenario-based experiences. The relaxation module featured neutral and calming virtual environments, such as an underwater world and natural mountain landscapes. Participants were encouraged to explore these environments freely while experiencing soothing audiovisual stimuli intended to induce relaxation and alleviate emotional tension. In the interactive scenario module, participants assumed the role of a “traveler,” guided by virtual companions, including a guiding star (“ShuoShuo”), a rabbit character (“MenMen”), and a collecting mouse (“Diandian”). Through voice guidance and preset tasks, participants interacted with the virtual environment using handheld controllers. Tasks included locating and selecting target objects (e.g., collecting “Starlight Fruit” or “Moon Moss”) and making choices based on simple calculations during simulated transactions. Each task had to be completed to advance the storyline and unlock subsequent activities.

Each intervention session lasted approximately 25 min and followed a standardized structure: adaptation and familiarization with the VR environment (about 5 min), relaxation training (about 10 min), and task-based interactive experiences (about 10 min). To ensure intervention fidelity, all participants received exactly the same VR content in the same order and duration. All intervention sessions were conducted according to a standardized protocol under the supervision of uniformly trained researchers, who had completed standardized training prior to the study.

#### 3.6.4 Adherence, modifications, and concomitant care

Adherence to the VR intervention will be monitored by recording attendance and completion of each session. Participants who miss a session will be rescheduled within the same week. The intervention is standardized; no modifications to the VR protocol will be made for individual participants. Participants in both groups may continue their prescribed antidepressant treatment as determined by their treating psychiatrist. No additional psychotherapies (e.g., cognitive-behavioral therapy) are permitted during the study period to avoid confounding. Any use of prohibited concomitant interventions will be documented.

### 3.7 Outcomes

The primary outcome of this study is the change in the 24-item Hamilton Depression Rating Scale (HAMD-24) (Wiebe et al., 2022) score from baseline (T0) to post-intervention (T1). Assessments will be conducted by trained evaluators who are blinded to group allocation.

Secondary outcomes include: 1. Anxiety symptoms, assessed using the Hamilton Anxiety Rating Scale-14 (HAMA-14) (Li Y. et al., 2025); 2. Self-reported depressive symptoms (Li X. et al., 2025), assessed using the Children’s Depression Inventory (CDI); 3. Cognitive function (Jia et al., 2021), assessed using the Montreal Cognitive Assessment (MoCA). All psychological assessments will be conducted at baseline (T0), post-intervention (T1), and follow-up (T2). 4. Heart rate variability (HRV) parameters (Shapiro et al., 2007; Kircanski et al., 2019), including SDNN, RMSSD, low-frequency power (LF), high-frequency power (HF), and the LF/HF ratio, will be measured as objective physiological outcomes. Electrocardiogram signals will be recorded for at least 5 min under resting conditions in a quiet environment, and both time-domain and frequency-domain analyses will be performed.

Although the HAMD was originally developed for adults, it has been widely used in clinical studies involving adolescents with major depressive disorder and has demonstrated acceptable reliability and validity in adolescent populations (Zhao et al., 2023). Previous studies have shown that HAMD scores are significantly correlated with other well-established measures of depression severity in adolescents, supporting its clinical utility in assessing symptom severity and treatment response (Croarkin et al., 2021; Li et al., 2023). The HAMD is routinely used in psychiatric clinical practice, and our team has extensive experience in its administration. The scale has good sensitivity to symptom change and facilitates comparisons with previous depression trials. To enhance the assessment of depressive symptoms and address potential developmental limitations, the CDI—a developmentally appropriate self-report measure for children and adolescents—will also be administered at all assessment time points. The combined use of a clinician-rated scale and a self-report measure is expected to provide a more comprehensive assessment of the severity of depressive symptoms.

The MoCA was selected as a brief, multidomain cognitive screening tool to assess cognitive domains commonly affected in depression, including attention, executive function, memory, and visuospatial ability. Although the MoCA was originally developed for adults, previous studies have demonstrated its acceptable feasibility and validity in adolescent populations (Stainton et al., 2025). In this study, the MoCA is not intended as a diagnostic tool for cognitive impairment but rather as a practical instrument for monitoring potential changes in overall cognitive functioning over time. Given its brevity, ease of administration, and multidomain structure, the scale is considered well-suited for repeated assessments among hospitalized adolescents.

### 3.8 Data collection and assessment schedule

Participants will be recruited from the Department of Psychiatry at the Affiliated Hospital of Henan Medical University. During the initial screening phase, potential participants and their legal guardians will be provided with detailed information about the study. Eligibility will be preliminarily assessed based on age, confirmed diagnosis of depressive disorder, and the absence of major physical illnesses or a history of epilepsy.

Participants who pass the preliminary screening will be invited to attend a face-to-face interview to further verify the inclusion and exclusion criteria. Written informed consent will be obtained from both participants and their legal guardians prior to enrollment.

Eligible participants will then be enrolled in the study and undergo baseline assessment (T0), followed by post-intervention assessment (T1) and follow-up assessment (T2). The detailed schedule of data collection is presented in Table 1.

**Table 1:** Timeline of data collection.

Item	Recruitment	Pre-treatment (TO)	Post-treatment (T1)	Follow-up (T2)
Screening	×			
Demographics	×			
Medical history	×			
Informed consent	×			
Randomization		×		
HRV		×	×	×
HAMD-24		×	×	×
HAMA-14		×	×	×
CDI		×	×	×
MoCA		×	×	×

HRV, Heart Rate Variability; HAMD-24, Hamilton Depression Rating Scale, 24-item; HAMA-14, Hamilton Anxiety Rating Scale, 14-item; CDI, Children’s Depression Inventory; MoCA, Montreal Cognitive Assessment.

### 3.9 Retention and follow-up

To promote participant retention and complete follow-up, participants will receive reminder calls or messages 1 day before each scheduled assessment. Efforts will be made to collect primary outcome data even from participants who discontinue the intervention, and these participants will be encouraged to complete the follow-up assessments. Reasons for dropout will be documented.

### 3.10 Data management

All study data will be managed using a unique identification coding system. Personal identifiable information will be stored separately from research data to ensure participant confidentiality.

Data will be maintained in a secure, password-protected electronic database, with access restricted to authorized members of the research team only. Data quality will be monitored regularly by designated research personnel to ensure accuracy and completeness.

### 3.11 Statistical analysis

All statistical analyses will be performed using SPSS (version 26.0), with two-sided tests and a significance level set at *p* < 0.05. Baseline characteristics will be summarized using descriptive statistics. Continuous variables will be presented as mean ± standard deviation (SD) or median (interquartile range), depending on the data distribution, while categorical variables will be expressed as frequencies and percentages. Comparability of baseline characteristics between groups will be assessed using independent-samples *t*-tests, Mann–Whitney *U* tests, chi-square tests, or Fisher’s exact tests, as appropriate.

The primary outcome measure (HAMD-24 score) will be analyzed using linear mixed-effects models (LMM) to account for repeated measurements over time. Treatment group (intervention vs. control), time (T0, T1, and T2), and the group-by-time interaction will be specified as fixed effects. A subject-specific random intercept will be included as a random effect to account for within-subject correlations across repeated assessments. The group-by-time interaction will be considered the main parameter of interest for evaluating the intervention effect. Baseline values of the corresponding outcome measure will be included as covariates in the model to improve statistical precision and control for potential baseline differences. Estimated marginal means, mean differences, 95% confidence intervals, and associated *p*-values will be reported.

Secondary outcome measures, including the HAMA-14, CDI, MoCA, and HRV indices, will be analyzed using similar linear mixed-effects models. Given the exploratory nature of these secondary outcomes, the results will be interpreted with caution and considered hypothesis-generating.

All efficacy analyses will follow the intention-to-treat (ITT) principle. Missing data will be handled using multiple imputation under the missing at random (MAR) assumption. The imputation model will include treatment allocation, demographic characteristics, baseline outcome values, and available follow-up measurements. Results from the multiply imputed datasets will be combined according to Rubin’s rules. Sensitivity analyses will be conducted to assess the robustness of the primary findings. Specifically, complete-case analysis and per-protocol analysis (including only participants who completed the intervention as planned) will be performed, and the results will be compared with those from the primary ITT analysis. For categorical outcome measures, between-group differences will be analyzed using chi-square tests or logistic regression models, as appropriate.

### 3.12 Safety monitoring and adverse events

Participants will be continuously monitored during each virtual reality session for potential VR-related adverse effects, including dizziness, nausea, headache, visual discomfort, eye strain, fatigue, and disorientation.

Before each VR session, participants will be asked about any physical discomfort that might affect their participation. During the intervention, trained researchers will continuously observe participants for signs of cybersickness or other adverse reactions. Immediately after each session, participants will be asked about the presence and severity of any VR-related symptoms using a structured symptom checklist and open-ended questions.

All adverse events will be recorded in a standardized case report form, including symptom type, onset time, duration, severity, actions taken, and outcome.

A VR session will be suspended or terminated if the participant requests to stop, reports moderate-to-severe discomfort (e.g., dizziness, nausea, headache, visual disturbances, or disorientation), or if the supervising researcher determines that continued participation may compromise safety. Participants who experience persistent or recurrent adverse reactions will be referred for clinical evaluation, and their continued participation in the intervention will be jointly reviewed by the study director and the attending physician.

## 4 Ethics and dissemination

### 4.1 Ethics approval

This study has been approved by the Institutional Review Board of Xinxiang Medical University (Approval No. XYLL-20250494) and the Ethics Committee of the Second Affiliated Hospital of Xinxiang Medical University (Approval No. XYEFYLL-(Research)-2025–116). Any protocol amendments will be submitted to both ethics committees for approval prior to implementation.

### 4.2 Informed consent

Trained research staff will obtain written informed consent from both the participants and their legal guardians after providing a thorough explanation of the study’s purpose, procedures, potential risks, and benefits. Consent will be obtained before any study-related procedures are performed. The informed consent form is provided as Supplementary File 1.

### 4.3 Safety monitoring and adverse events

All adverse events (AEs) and serious adverse events (SAEs) will be recorded from enrolment through the follow-up period. Participants will be asked at each visit about any discomfort or unexpected symptoms. AEs will be assessed for severity, causality, and expectedness. SAEs will be reported to the ethics committees and the sponsor within 24 h. The principal investigator is responsible for managing AEs and ensuring participant safety. No independent data monitoring committee was established, as the trial involves a low-risk, short-term intervention and is monitored jointly by the investigators and the institutional ethics committees.

### 4.4 Data access and confidentiality

Only the principal investigator and authorized statisticians will have access to the final de-identified dataset. All personal identifiers will be stored separately from research data in a password-protected, encrypted database. No contractual agreements limit access to the data for investigators.

### 4.5 Dissemination policy

The results of this study will be submitted for publication in peer-reviewed journals and presented at national and international conferences. The findings will be shared with participants via a plain-language summary distributed through the clinical team upon request. There are no publication restrictions.

## 5 Discussion

One of the potential strengths of this study is its ability to explore the multiple pathways through which a virtual reality-based intervention may influence depressive symptoms in adolescents. Although the precise mechanisms remain to be fully elucidated, several complementary processes may contribute to the therapeutic effects. First, the intervention incorporates interactive task-oriented activities and goal-directed engagement within an immersive virtual environment. These features may promote behavioral activation, a well-established therapeutic principle in depression treatment. By encouraging active participation, task completion, and positive reinforcement, the intervention may help counteract the withdrawal, reduced activity, and diminished motivation commonly observed in depressed adolescents. Second, the immersive nature of virtual reality may facilitate emotion regulation. Relaxation-oriented environments, such as underwater and forest landscapes, are designed to reduce psychological stress and provide opportunities for emotional soothing. Furthermore, guided interactions with virtual companions may enhance adaptive emotional processing and reduce negative emotional reactivity. Improvements in these processes may be reflected in reductions in depressive and anxiety symptoms as measured by the HAMD-24, CDI, and HAMA-14. Third, the intervention may influence cognitive functioning by engaging participants in sustained attention, problem-solving, and interactive decision-making tasks embedded within the virtual scenarios. Previous research has shown that adolescents with depression often exhibit impairments in attention, executive function, and cognitive flexibility. By involving participants in structured cognitive activities, the VR intervention may help improve cognitive performance, an aspect that will be explored through the MoCA. Finally, virtual reality may exert physiological regulatory effects by modulating autonomic nervous system activity. According to the neurovisceral integration model, depression is associated with impaired autonomic flexibility and reduced heart rate variability. Immersive relaxation experiences may reduce stress-related sympathetic activation and promote parasympathetic recovery, potentially leading to improvements in HRV indices. Thus, the inclusion of heart rate variability measurement offers an opportunity to examine whether psychological improvements are accompanied by objective changes in autonomic regulation.

Recent research further suggests that the therapeutic value of virtual reality may extend beyond symptomatic distraction to involve broader psychological and physiological regulatory processes. For example, studies have reported that (Wei et al., 2026) VR-based exercise and extreme sports interventions can improve emotional well-being, reduce perceived stress, and enhance psychological resilience in vulnerable adolescent populations. Similarly, immersive VR experiences have been associated with reduced anxiety and stress responses in individuals with social anxiety disorder (Wang Z. et al., 2025). These findings indicate that virtual environments may promote psychological adaptation through active engagement, experiential learning, and positive emotional experiences, rather than merely passive observation. Although the intervention in this study differs from VR-based exercise and extreme sports paradigms, several underlying mechanisms may be shared. Both approaches rely on immersive engagement, interactive participation, and the provision of novel experiences that may temporarily divert attention from maladaptive cognitive patterns while encouraging adaptive emotional processing. For adolescents with depression, who often exhibit reduced motivation, social withdrawal, and persistent negative self-focused thinking, such immersive, goal-oriented experiences may facilitate behavioral activation and emotional engagement, thereby contributing to symptom improvement. From a broader public health perspective, emerging evidence supports VR-based interventions as an accessible and engaging non-pharmacological approach with the potential to promote mental health and alleviate psychological distress (Wang L. et al., 2025). Compared with these previous studies, the present study differs in several important respects. First, it specifically focuses on adolescents with a clinical diagnosis of major depressive disorder, a population that remains understudied in VR intervention research. Second, the intervention in this study combines immersive relaxation environments with interactive tasks designed to promote emotion regulation and behavioral engagement. Third, the study includes not only symptom-based assessments but also cognitive and physiological outcome measures, thereby allowing for the exploration of potential mechanisms underlying treatment effects. By integrating affective, cognitive, and autonomic outcome domains within a randomized controlled design, this study may contribute to a more comprehensive understanding of how VR interventions affect adolescent depression.

This study also has several limitations. First, it is a single-center study with a relatively limited sample size, which may affect the generalizability of the findings. Second, due to the nature of the intervention, complete blinding of participants is difficult to achieve, and non-specific effects may be present. Third, an active control condition was not included. The control group received usual care, while the intervention group received usual care plus the VR intervention. Therefore, the observed between-group differences cannot be attributed solely to the immersive VR technology itself, but may also be influenced by factors such as increased engagement, novelty effects, expectancy effects, or more frequent interactions with research staff. Future studies incorporating an active control condition, such as a non-immersive digital intervention or an alternative psychosocial activity, would help clarify the specific effects of immersive VR technology. Fourth, the study included participants aged 10–19 years, covering a relatively broad developmental period. Adolescence is characterized by significant cognitive, emotional, and neurobiological changes, and the effects of the VR intervention may vary across developmental stages. Although randomization can balance age distribution between groups, the current sample size does not provide sufficient power for well-powered subgroup analyses. Therefore, age-related heterogeneity should be considered when interpreting the results. Fifth, although the VR intervention was delivered according to a standardized protocol, no formal treatment fidelity assessment was conducted. Differences in engagement, intervention adherence, or implementation consistency may not have been fully captured. Future studies should consider incorporating structured monitoring procedures, such as intervention checklists, session completion records, and independent fidelity ratings, to further strengthen internal validity.

Finally, participants will be followed up for 3 months after the intervention, with follow-up assessments conducted at 1, 2, and 3 months after discharge. The primary aim of the follow-up is to assess the short- to intermediate-term maintenance of treatment effects on depressive symptoms, autonomic function, cognitive function, and related psychological outcomes. The three-month follow-up period was chosen based on both methodological and practical considerations. On the one hand, this study aims to examine the immediate, short-term, and intermediate-term effects of the VR intervention rather than long-term relapse prevention. On the other hand, the study population consists of hospitalized adolescents with depression; longer follow-up periods may be associated with increased dropout rates as participants return to school or community settings after discharge. Therefore, this study is not intended to determine long-term sustainability of treatment effects, relapse rates, or relapse prevention outcomes beyond the three-month observation period. Any benefits observed during the 3-month follow-up should be interpreted as evidence of short- to intermediate-term maintenance rather than long-term efficacy. Future studies with longer follow-up periods (e.g., 6–12 months) are needed to assess the durability of treatment effects and their potential impact on relapse prevention.
