# Supplementary material for: Effectiveness of virtual reality–based intervention for adolescent depressive disorder: a randomized controlled trial protocol
Source: Front Psychol. 2026 Jul 7;17:1845011. doi: 10.3389/fpsyg.2026.1845011 (PMC13385189; doi:10.3389/fpsyg.2026.1845011)
Supplement: Supplementary file 1 [file Supplementary_file_1.pdf]

Legal Guardian Consent

Name of Parent/Legal Guardian:

Relationship to Participant:

Signature:

Date:

Dear Sir/Madam,

You are invited to participate in the study titled Intervention Study on the Rehabilitation Effect of Virtual Reality Technology in Adolescents with Depressive Disorder. Before you decide whether to participate in this study, please read the following information carefully. It will help you understand the purpose, procedures, duration, potential benefits, risks, and discomforts associated with the study.

1. Research Background

Depressive disorder is a condition caused by multiple factors, characterized by and persistent depressive symptoms as its main clinical feature. Its core symptoms include persistent low mood, loss of interest and energy, and it typically begins in late adolescence or early adulthood. The incidence of depressive disorder has been rising, and currently more than 300 million people worldwide are affected, imposing a heavy social and economic burden and reducing the quality of life of those affected. According to the Global Burden of Disease, Injuries, and Risk Factors Study 2019, depressive disorder is one of the two most disabling mental disorders and ranks among the top 25 causes of global burden. Adolescent mental health is crucial for the vigorous development of the country's future and is an important part of the "Healthy China" initiative. With social development and progress, adolescents in China are under multiple pressures from academics, social interactions, and personal development, leading to a gradual increase in the incidence of mental health problems. Adolescent depressive disorder often presents with emotional instability, irritability, weight loss, physical discomfort, school refusal, and other clinical features, with significant mood fluctuations and a higher risk of impulsive and suicidal behaviors. According to the Blue Book of Mental Health in China (2021–2022), the detection

rate of depressive disorder among Chinese adolescents is 14.8%. For adolescents, who are in a critical period of neurodevelopment, their cognitive control, emotion regulation, and social cognition are still unstable, making them more prone to negative automatic thoughts and rumination patterns, which can maintain or worsen depressive symptoms and affect academic and social functioning. Stressors in school and family environments, digital lifestyles, and peer interaction characteristics may further amplify these risks.

In psychiatry, traditional treatment tools are mainly limited to interpersonal psychotherapy and pharmacotherapy, whereas VR can provide various types of stimulation to help patients more safely. Immersive VR is also considered a potentially revolutionary psychological intervention tool. Virtual Reality (VR) technology, also known as virtual simulation technology, is a cutting-edge form of human–computer interaction. Through computer systems and specialized software, it constructs highly realistic virtual environments, allowing users to immerse themselves in simulated situations and obtain near-real experiences. As an effective adjunctive treatment tool, VR technology is widely used in medicine, including medical education and training, surgical simulation and planning, clinical treatment and diagnosis, rehabilitation, and mental health treatment. VR technology can provide novel solutions to improve medical quality and patient outcomes. With technological advances and increasing public awareness of VR, its application in medicine is expanding. Clinical studies have made significant progress in treating post-traumatic stress disorder (PTSD), phobias, and anxiety disorders. For example, Hong et al. used VR to simulate heights and help patients with acrophobia reduce fear. VRET studies for social anxiety primarily aim to relieve excessive anxiety during public speaking. Kampmann, Hofmann, Anderson, and others used VRET to help individuals with social anxiety overcome speech anxiety, with relatively stable treatment effects maintained during 4–6 years of follow-up. VRET research for PTSD has focused on war, traffic accidents, and terrorist attacks. Kuntze et al. first used VR technology to treat substance use disorders (SUD), presenting virtual stimuli to activate patients' cravings. Riva et al. treated patients with eating disorders by creating five scenarios

addressing abnormal eating behaviors. The clinical potential of VR is clearly supported by these results. Although a meta-analysis by Cieślik et al. supports the effectiveness of VR treatment for various mental disorders in adults, its application in adolescent mental health remains severely insufficient. A systematic review and meta-analysis of VR technology for treating depression in adolescents and young adults indicate that VR technology shows promise in treating adolescent depression by building immersive, highly interactive virtual situations that help adolescent patients with emotion regulation and cognitive behavioral training.

Cognitive Behavioral Therapy (CBT), based on cognitive psychology and behavioral science theories, improves emotional and behavioral problems by identifying and adjusting maladaptive cognitive and behavioral patterns. Clinical studies have confirmed that CBT has significant efficacy for adolescent depressive disorder and is recommended as a first-line psychological treatment by authoritative bodies such as the American Psychological Association (APA) and the UK National Institute for Health and Care Excellence (NICE). On this basis, the integration of VR and CBT (VR-CBT) enhances contextualized cognitive restructuring, exposure and behavioral activation, attention bias modification, and generalization of emotion regulation skills. It provides controllable triggering cues and graded task difficulty, facilitating systematic desensitization and skill transfer. Its high immersion and immediate feedback increase engagement and adherence, reducing adolescents' sense of alienation from traditional talk therapy. It objectifies cognitive processes (e.g., executive function, working memory, attention inhibition) through interactive tasks, promoting generalization to real-life situations. In recent years, with VR technology development, VR-CBT has been widely used in mental health fields, changing patients' maladaptive cognitions in multiple ways. Compared with traditional CBT, VR-CBT has also been shown to effectively treat patients with depressive disorder.

Heart Rate Variability (HRV) refers to the variation between consecutive heartbeats, reflecting adaptation to environmental changes in pulse length. HRV is influenced by parasympathetic activation (including the vagus nerve, which slows heart rate) and sympathetic activation (which accelerates heart rate). Clinically, HRV

was initially used to assess the severity of cardiovascular disease (CVD) and then extended to psychiatric disorders (e.g., CVD patients with comorbid mental disorders). However, increasing evidence shows that psychiatric patients without CVD also exhibit abnormal HRV. Given the high comorbidity between depressive and anxiety disorders, and HRV has been theoretically considered a transdiagnostic biomarker of psychopathology, HRV abnormalities may be associated with transdiagnostic internalizing symptoms. HRV is a physiological characteristic that reflects autonomic nervous system function. Recently, HRV has been increasingly used in mental health, stress coping, and autonomic dysregulation, and this biomarker is considered to have great potential for early identification and monitoring of mental health problems. Moreover, internalizing symptoms often emerge during adolescence.

In summary, depressive disorder has become a core component of the global disease burden. Adolescents, as a vulnerable group, are more susceptible to adverse environmental and psychosocial factors due to their cognitive and emotional regulation vulnerabilities, leading to onset or relapse. VR technology provides a new platform for delivering CBT, enabling the reconstruction of adolescents' cognitive patterns and training of emotion regulation skills in safe, immersive, and highly contextualized environments, thereby enhancing efficacy and scalability. However, the application and validation of VR-CBT in adolescent depressive disorder remain very limited. Therefore, this study will focus on the innovative combination of VR intervention and HRV indicators, aiming to provide a more comprehensive, physiologically supported nursing practice protocol for the "healing" of adolescent depressive disorder. This has both important theoretical significance and guiding value for promoting innovation in adolescent mental health intervention models, optimizing nursing practice, and improving the accessibility and effectiveness of the psychosocial service system.

2. Study Objectives

（1） To evaluate the effect of VR scene intervention on clinical symptoms of adolescent depressive disorder.

（2） To analyze changes in HRV indicators during VR intervention, explore their

correlation with depression severity, and clarify the mechanism by which VR intervention affects autonomic nervous system function.

（3）To verify the comprehensive efficacy and overall advantages of VR in relieving depressive symptoms and improving cognitive function.

3. Study Content

This study aims to construct a CBT-based VR intervention protocol and verify its effectiveness on clinical symptoms of adolescent depressive disorder through a randomized controlled trial. First, through systematic literature search and evidence-based analysis, we will comprehensively review the core CBT intervention elements for adolescent depressive disorder and the application modes of VR technology in psychotherapy, thereby forming a preliminary theoretical framework and draft of the VR-CBT intervention protocol. During the implementation phase, a randomized controlled trial design will be adopted. Using cluster sampling, eligible adolescents with depressive disorder will be selected and randomly assigned to an experimental group or a control group using a random number table. The experimental group will receive VR-CBT intervention for 4 weeks (12 sessions) in addition to routine medication and nursing care; the control group will receive only routine medication and nursing care. At baseline (T0), immediately after intervention (T1), and at follow-up (T2), participants will complete the General Information Questionnaire, Hamilton Depression Rating Scale (HAMD), Hamilton Anxiety Rating Scale (HAMA), Children's Depression Inventory (CDI), and Montreal Cognitive Assessment (MoCA) to collect clinical symptom and cognitive function data. HRV indicators at rest will also be collected using specialized equipment to quantitatively evaluate the psychophysiological effects of the intervention. Finally, this study will comprehensively evaluate the effectiveness, acceptability, and applicability of the VR-CBT protocol, providing comprehensive empirical evidence for optimizing intervention strategies and advancing clinical rehabilitation nursing applications.

4. Study Methods

We will review relevant literature to understand the current status and shortcomings of VR technology application in adolescent depressive disorder, and

preliminarily formulate the study population and intervention protocol based on existing research.

This study is planned to be conducted from December 2025 to June 2026. Using cluster sampling, participants will be recruited from adolescents with depressive disorder admitted to the Child and Adolescent Psychiatric Department of Henan Provincial Mental Hospital. Participants will be randomly assigned to an experimental group or a control group using a random number table.

Inclusion criteria:

① Age 10–19 years;

② Diagnosed by a psychiatrist as meeting the diagnostic criteria for depression according to the ICD-10;

③ Receiving stable medication;

④ Normal reading and writing abilities, no communication impairment;

⑤ Patient and family members provide informed consent and voluntarily participate.

Exclusion criteria:

① Current or past history of organic brain disease or severe traumatic brain injury, personal or family history of epilepsy;

② Severe cardiac, hepatic, or renal dysfunction;

③ Severe physical illness; history of substance dependence or abuse (alcohol, cocaine, drugs, etc.);

④ Comorbid other mental disorders such as schizophrenia, bipolar disorder (hypomanic or manic episodes);

⑤ History of motion sickness or vertigo, or any active nausea/vomiting.

Sample size calculation: A pre-survey will be conducted among adolescent depressive disorder patients admitted to the hospital from December 2025 to June 2026. Considering potential data loss and invalid questionnaires, and based on the study's objectives and methods, the formula for comparing two independent sample means will be used: $n_1 = n_2 = 2\left[\frac{(\mu_\alpha+\mu_\beta)}{\delta/\sigma}\right] + \frac{1}{4}\mu_{\alpha^2}$, where n1、n2are the sample sizes per group,

σ is the estimated overall standard deviation，δ is the clinically meaningful difference in scores between the two groups, μ_α corresponds to the type I error probability α, and μ corresponds to the type II error probability β. Based on relevant literature, the calculated sample size was 32 per group. Adding a 10% attrition rate, the final sample size was set at 36 per group.

Intervention team:

The team consists of one child and adolescent psychiatrist, one rehabilitation therapist, one clinical psychotherapist, one VR technology expert, two senior psychiatric nurses, and two graduate students. The psychiatrist is responsible for diagnosis, eligibility determination, and timing of intervention. The rehabilitation therapist reviews intervention content, assesses participants' condition, and selects appropriate VR scenes. The psychotherapist monitors the quality of the intervention environment and protocol and conducts scale assessments. The senior nurses ensure quality control and participate in each session. The VR expert builds virtual scenes, imports materials, maintains equipment, and adjusts parameters. The graduate students develop the intervention protocol, write scripts, determine the intervention cycle and frequency, and implement the protocol after revisions based on feedback from clinicians and experts.

Intervention scenes:

Relaxation scenes (also neutral scenes) include underwater world and mountain-river forest. In the VR scenes, participants will act as a "traveler" accompanied by a series of procedurally generated, empathetic virtual companions (e.g., guiding star "Shuoshuo," stamping rabbit "Menmen," collecting mouse "Diandian"). These virtual characters guide participants through gentle voice commands and preset tasks. Participants must use handheld controllers to locate and select target objects (e.g., picking "starlight fruit," "moonlight moss"), perform simple arithmetic selections (e.g., trading at the "Stardust Shop"), and respond to task requirements to advance the narrative to the next stage.

Intervention equipment:

Laptop computer, VR headset (model: HTC Vive Focus Pro), VR controllers, and CBT-based VR scenes developed by the research team.

Intervention schedule:

The intervention consists of three stages. Stage 1: participants adapt to the head-mounted VR device and learn to use the controllers. Stage 2: participants become familiar with the VR intervention format and scenes. Stage 3: participants engage in the full VR intervention. The intervention lasts 4 consecutive weeks, with 12 sessions total (3 sessions per week, 25 minutes per session).

Intervention methods:

Participants meeting the inclusion/exclusion criteria will be randomly assigned to two groups using a random number table: routine medication group and VR + routine medication group. Before the intervention, both groups will complete the General Information Questionnaire to collect baseline data. HRV will be recorded, followed by completion of HAMD, HAMA, CDI, and MoCA; these data will serve as baseline assessment indicators.

Intervention group:

In addition to routine medication, participants will receive VR intervention training. Before each session, participants will be informed of the precautions and procedures. In the virtual scenes, participants must follow instructions given by virtual characters, using handheld controllers to position a cursor on targets or answer questions to proceed. Given the potential for VR-induced motion sickness, a “desensitization protocol” will be applied in the week before the intervention (gradually increasing game time based on individual differences) until the participant tolerates it. The design follows a high-frequency, short-cycle pattern based on effective studies in the literature: total intervention period 4 weeks, frequency 3 times/week, total 12 sessions. Each session lasts 25 minutes: 10 minutes of intervention, followed by 5 minutes of relaxation in a VR scene, then another 10 minutes of intervention.

Control group:

Participants will receive SSRIs at appropriate doses based on symptom severity

for 4 weeks.

After the intervention, the differences between the experimental group (4 weeks of VR + medication) and the control group (routine medication) will be compared to analyze the effect of long-term intervention on depressive symptoms. Subsequently, telephone follow-ups will be conducted at 30, 60, and 90 days after discharge to compare depressive symptoms between the two groups.

5. Benefits of Participation

If you agree to participate in this study, your child will receive free professional VR-CBT rehabilitation intervention for 4 weeks (12 sessions), including use of the HTC Vive Focus Pro VR headset and the research team's customized immersive rehabilitation scenes, at no cost. During the study, three multi-dimensional assessments will be provided free of charge (pre-intervention, post-intervention, and follow-up), including depression/anxiety symptom assessment (HAMD, HAMA, CDI), cognitive function assessment (MoCA), and HRV physiological indicator measurement. Participants may benefit from receiving the VR intervention; however, therapeutic benefit cannot be guaranteed. A team consisting of a psychiatrist, clinical psychotherapist, and rehabilitation therapist will monitor the participant's status throughout the intervention and adjust the protocol as needed. After discharge, free telephone follow-ups will be conducted, during which professionals will answer rehabilitation questions and provide targeted guidance.

6. Right to Refuse or Withdraw

You may choose not to participate in this study, or you may withdraw at any stage without giving any reason. Your child's medical treatment and rights will not be affected. However, data collected before withdrawal will be processed legally, and if such data have already been integrated into the research project and cannot be removed due to cost or other practical reasons, they may continue to be used in the study under the premise of protecting your privacy. Once you decide to participate, please sign this informed consent form. Before entering the study, the physician will screen the patient and you to confirm eligibility.

7. Privacy and Confidentiality

This study does not collect any personal information from caregivers; all data collection and recording are limited to the participating adolescent patients. The patient's name, gender, and other personally identifiable information will be replaced with codes or numbers and strictly kept confidential. Only the research-related medical staff can access the patient's research data. Study results may be published in journals, but no personally identifiable information of the patient will be disclosed.

If you agree to participate in this study, the patient's medical records may be reviewed only by personnel from the research sponsor, relevant regulatory authorities, or the independent ethics committee to verify the proper conduct of the study, and such reviews will strictly follow confidentiality principles. By signing this informed consent form, you agree to allow the aforementioned parties to review the patient's relevant research records (which do not involve caregiver personal information).

All electronic data will be stored on password-protected computers accessible only to authorized study personnel. Paper records will be kept in locked cabinets within the research office.

8. Informed Consent Signature

If you fully understand the content of this research project and agree to participate, you will sign this informed consent form in duplicate: one copy for the researcher and one for the participant/subject.

Project Title: Intervention Study on the Rehabilitation Effect of Virtual Reality Technology in Adolescents with Depressive Disorder

Consent Statement by the Subject:

I confirm that I have read and understood the informed consent form for this study, that potential issues and their solutions have been explained to me, and that I have had the opportunity to ask questions.

I understand that participation is voluntary and that refusal to participate will not harm any rights of the patient or myself.

I have been informed that the physicians involved in this study, the responsible personnel, and the hospital's ethics committee have the right to review the research records and medical records. I agree that such personnel may directly access the

research records and understand that such information will be handled confidentially.

I agree to participate in this study.

Full name of participant: ________________

Date: ________ (Year/Month/Day)

The following section to be completed by the medical staff obtaining informed consent:

Investigator's declaration: I confirm that I have explained and discussed the nature, purpose, requirements, and potential risks of this study with the patient, and I have ensured that a copy of this informed consent form has been provided to the subject for retention.

Full name of investigator: ________________

Date: ________ (Year/Month/Day)
